# Microscopical Analysis of Autofluorescence as a Complementary and Useful Method to Assess Differences in Anatomy and Structural Distribution Underlying Evolutive Variation in Loss of Seed Dispersal in Common Bean

**DOI:** 10.3390/plants12112212

**Published:** 2023-06-03

**Authors:** Ana M. Santos, Ana M. González, Juan De Dios Alche, Marta Santalla

**Affiliations:** 1 Centro de Instrumentación Científica, University of Granada, 18003 Granada, Spain; asantos@ugr.es; 2 Grupo de Genética del Desarrollo de Plantas, Misión Biológica de Galicia—Consejo Superior de Investigaciones Científicas (MBG-CSIC), 36080 Pontevedra, Spain; amgonzalez@mbg.csic.es; 3 Estación Experimental del Zaidín, CSIC, 18008 Granada, Spain; juandedios.alche@eez.csic.es; 4 Instituto Universitario de Investigación en Olivar y Aceites de Oliva (INUO), Universidad de Jaén, 23071 Jaén, Spain

**Keywords:** pod, anatomy, microscopy, cell wall, *Phaseolus vulgaris*, legume, autofluorescence

## Abstract

The common bean has received attention as a model plant for legume studies, but little information is available about the morphology of its pods and the relation of this morphology to the loss of seed dispersal and/or the pod string, which are key agronomic traits of legume domestication. Dehiscence is related to the pod morphology and anatomy of pod tissues because of the weakening of the dorsal and ventral dehiscence zones and the tensions of the pod walls. These tensions are produced by the differential mechanical properties of lignified and non-lignified tissues and changes in turgor associated with fruit maturation. In this research, we histologically studied the dehiscence zone of the ventral and dorsal sutures of the pod in two contrasting genotypes for the dehiscence and string, by comparing different histochemical methods with autofluorescence. We found that the secondary cell wall modifications of the ventral suture of the pod were clearly different between the dehiscence-susceptible and stringy PHA1037 and the dehiscence-resistant and stringless PHA0595 genotypes. The susceptible genotype had cells of bundle caps arranged in a more easily breakable bowtie knot shape. The resistant genotype had a larger vascular bundle area and larger fibre cap cells (FCCs), and due to their thickness, the external valve margin cells were significantly stronger than those from PHA1037. Our findings suggest that the FCC area, and the cell arrangement in the bundle cap, might be partial structures involved in the pod dehiscence of the common bean. The autofluorescence pattern at the ventral suture allowed us to quickly identify the dehiscent phenotype and gain a better understanding of cell wall tissue modifications that took place along the bean’s evolution, which had an impact on crop improvement. We report a simple autofluorescence protocol to reliably identify secondary cell wall organization and its relationship to the dehiscence and string in the common bean.

## 1. Introduction

The common bean (*Phaseolus vulgaris*) is a typical dry dehiscent fruit of the Fabaceae family, which is the third largest flowering plant family. The fruit dehiscence character has been regarded as a critical strategy in the domestication process due to its importance for the propagation of offspring in wild plants, but it is also a major cause of yield loss [1,2,3]. The phenotypic dehiscence variation in the common bean has increased considerably with domestication and under particular environmental conditions [4,5]. Pod dehiscence is associated with pod fibre content in legumes [6,7,8]. Indeed, while dry beans usually have dehiscent pods, snap or green beans are completely indehiscent (stringless varieties) [7], as they no longer have fibres in the pod sutures (string) and walls [9,10]. In beans and other legumes such as the cowpea, selection for indehiscence is accompanied by the edibility (pod softness/tenderness) of immature pods, indicating that pod fibre content and pod tenderness are genetically correlated traits [11,12,13]. For this reason, dehiscence has been extensively studied as a critical step for crop improvement in the domesticated common bean [14,15].

Most anatomical studies on the dehiscence process have been performed on Arabidopsis. Members of the Brassicaceae family produce a silique, a dehiscent elongated fruit that forms valve margins with two narrow layers spanning the length of the fruit. In one layer, cell separation occurs (the adjacent non-lignified cells of the replum), and in the other, lignified cells provide the necessary tension to open the silique; collectively, both are referred to as the dehiscence zone [16,17]. During the dehiscence process, the innermost layer of the valve, called the “endocarp a” layer, undergoes programmed cell death and starts to disintegrate, while the other region, the “endocarp b” layer, goes through cell wall lignification [18]. This increased lignin deposition of the “endocarp b” layer provides tension within the silique, causing the opening of the fruit and the release of the seeds, and it has been associated with dehiscence susceptibility [19,20]. The increased vascular tissue and the decreased cell wall degradation within the dehiscence zone have both been associated with dehiscence resistance [21]. Brassica and leguminous plants (such as the common bean) have multiple similarities in terms of their anatomical mechanisms of fruit dehiscence, as we show below, which necessitate further study.

The legume pod is composed of a single carpel fused at both sides, in contrast to the two carpels of the silique in brassicas. The valves of the pods are connected along the ventral and dorsal sutures, and the dehiscence zone (DZ) is located along both sutures, where the vascular bundles develop thick walls, and the ending arrangement is called a “bundle cap” (BC). This DZ terminates at the fibre cap cells (FCCs) at the border between the bundle cap and the mesocarp of the wall pod [22,23]. Pod dehiscence is a consequence of the weakening of the dorsal and ventral DZs, and the opening of the pod usually occurs on the dorsal side of the pod because the FCCs are the only structures connecting the valve edges at the dorsal side [24]. Despite the lack of a replum, the DZ is structurally similar to that of Arabidopsis fruits, and it shows two cell layers, namely, a non-lignified layer and a lignified separation layer, along the pod between both valves [22,25]. In legumes, studies aiming to understand the mechanisms of pod dehiscence have been conducted in alfalfa (*Medicago sativa*), birdsfoot trefoil (*Lotus corniculatus*), common vetches (*Vicia sativa*), narrow-leafed lupin (*Lupinus angustifolius*), chickpeas (*Cicer arietinum*), peas (*Pisum sativum*), lentils (*Lens culinaris*), faba beans (*Vicia faba*), soybeans (*Glycine max*), cowpeas (*Vigna unguiculata*), and common beans [19,21,26,27,28,29,30,31]. These studies have suggested that histological differences in the DZ between non-dehiscent and dehiscent phenotypes are mainly associated with the degree of lignin deposition of the FCCs, located at the junctions between the valves, and the heavily thickened secondary cell walls, which greatly strengthen the connection between both valves and prevent dehiscence. The other decisive driver of pod dehiscence is directly related to tension developed in the cells of the inner sclerenchymatous layer because of dehydration, which is associated with the contents of cellulose, hemicellulose, and lignin components and/or the structure of the pod wall, and is also associated with changes in the orientation and geometry of the cells, as well as unequal expansion and contraction of the layers [12,16]. In the common bean, a lignified wall fibre layer that is thicker than the vascular bundle suture layer is associated with dehiscence, suggesting similarities with the lignification pattern and secondary cell wall thickening of the dehiscent fruit of Arabidopsis and with valve lignification of the dehiscent fruit in soybeans [32,33]. Other authors [9] have observed a high percentage of fibre cells (lignified and heavily thickened) in dehiscent (stringy) fruit and a predominance of wood cells (lignified but not thickened) in indehiscent (stringless) fruit along both sutures. In brassicas, dehiscence initiates either at the base or at the apex of the fruit [34] and stretches across DZ until the valves are completely separated [35]. Legume species differ in the suture of the pod for dehiscence initiation. Thus, pod dehiscence of the vetch legume first occurs in the ventral suture [36]. In soybeans, it usually starts on the dorsal side of the pod [24,37,38], while in the common bean, the ventral suture is also critical to pod dehiscence. Further investigations on pod tissues, including the sclerenchymatous dorsal and ventral sutures as key traits for pod dehiscence, can help us to fully understand the common bean pod anatomically and morphologically.

One of the major challenges is to enhance our capacity to characterize the spatial conformation of lignin of the secondary cell wall, since lignin confers hydrophobicity and rigidity to the cell wall, and lignin contents and their localization have been associated with dehiscence [39]. Studies on global lignin-containing tissue detection and function have exploited different physical and histochemical methods, in addition to the natural autofluorescence of lignin [40] and basic Fuchsin, Auramine, Acriflavine [41,42], Phloroglucinol–Wiesner [43], and Safranin/Alcian blue staining [44]. However, many of these procedures are time-consuming and affect tissue integrity. With the continuous advance in imaging techniques, it is now possible to adjust the procedures to achieve efficient and highly resolute methodologies, which are more suitable for genomics approaches, in order to analyse a large number of samples. In this research, we studied two contrasting genotypes for dehiscence and the presence of pod string by comparing different histochemical methods with autofluorescence, in order to gain better structural knowledge of the tissues involved at different stages of pod development. 

## 2. Results

### 2.1. Histological Characterization of the Dehiscence Zone in the Common Bean Pod

**Figure 1:**
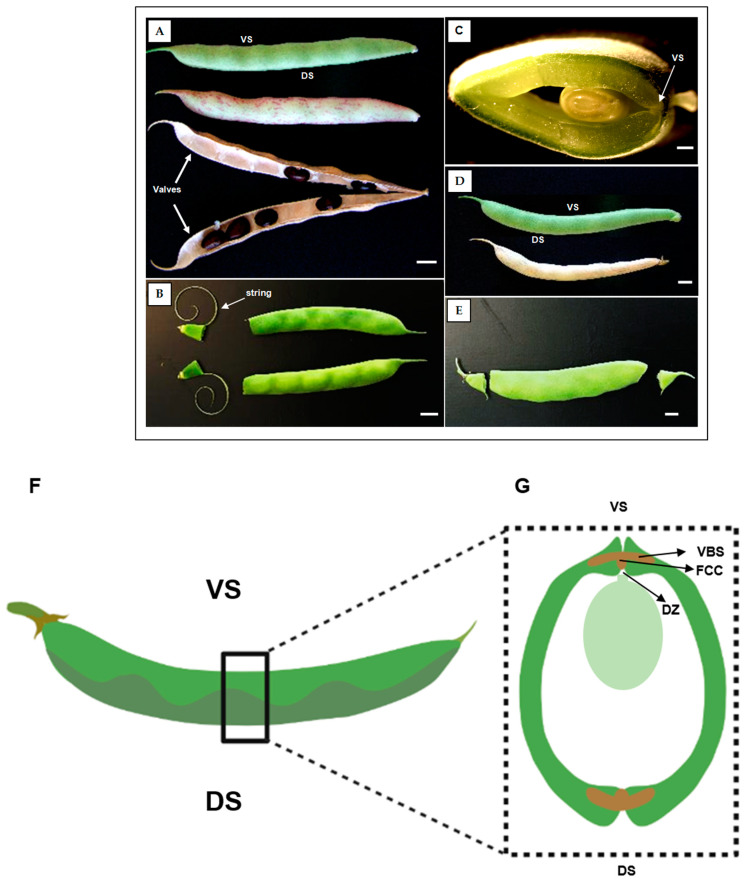
Phenotypes for pod dehiscence and string in cultivated and wild common bean. Pods of the dehiscence-susceptible and stringy genotype PHA1037 (**A**–**C**) and the dehiscence-resistant and stringless genotype PHA0595 (**D**,**E**) at 30 days after anthesis (DAA). Suture string is present in the ventral suture of PHA1037 pods (**B**,**C**). Graphical structure of a lateral view (**F**) and transverse section (**G**) of a common bean pod. Ventral suture: VS, dorsal suture: DS, vascular bundle sheath: VBS, dehiscence zone: DZ, and fibre cap cells: FCCs. The figure is modified from [14]. Scale bar: 1 cm.

The common bean pod is composed of a single carpel that encloses the seeds, with two sutures; namely, a dorsal and a ventral suture. To explore the changes that take place during the pod maturation process and its relationship with the string and dehiscence, fully mature green pods (R8 stage) and dried physiological senescent brown pods (R9 stage) [45] were sampled from two contrasting genotypes and prepared for microscopy. The morphologies of both genotypes are shown in Figure 1A–E, and micrograph images of the area around the dorsal and ventral sutures of the pod are shown in Figure 1F,G.

**Figure 2:**
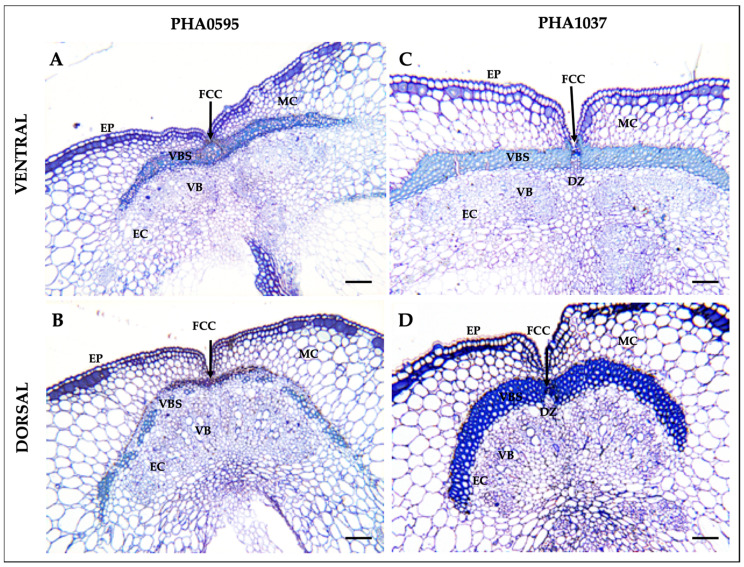
General overview of Toluidine blue O-stained pod sutures in transverse sections of common bean. Semithin epoxy-embedded sections (1 μm thick) of 40 days after anthesis (DAA) pods (**A**–**D**). Transverse pod sections of the ventral suture (**A**–**C**) and dorsal suture (**B**,**D**) of the dehiscence-resistant and stringless genotype PHA0595 (**A**,**B**) and dehiscence-susceptible and stringy genotype PHA1037 (**C**,**D**). Ventral suture: VS, dorsal suture: DS, bundle cap: BC, dehiscence zone: DZ (in PHA1037 genotype), fibre cap cells: FCCs, vascular bundles: VB, vascular bundle sheath: VBS, epidermis: EP, endocarp: EC, mesocarp: MC. Scale bars: 100 μm.

In order to gain an overview of the cell wall morphology during pod development, we first used Toluidine Blue O (TBO) staining (Figure 2A–D). Micrographs of the transverse sections revealed that the common bean pod wall (pericarp) follows the common arrangement and structure described in legume crops (Figure 2A–D) [46,47]. The exocarp consists of a monolayer epidermis (EP), while the mesocarp (MC) is arranged in several layers of parenchymal cells. The endocarp (EC) comprises a strongly lignin-thickened sclerenchyma at the dorsal and ventral sutures, with two lateral branches extending into the seed coats, and a vascular bundle (VB) containing the xylem and phloem tissues. Both the exocarp and mesocarp are rich in pectins, as identified by metachromatic staining with Toluidine blue (Figure 2A–D). The DZ was observed in both sutures between the valves of the pod as a narrow band of valve margin cells between vascular bundle sheaths (VBSs), which connect the two valves together through the FCCs, and it is one of the important structures of legume pods (Figure 2A–D). TBO staining also revealed clear differences between genotypes regarding the sheath lignified area in the VBS, which was much more pronounced in the dehiscent and stringy PHA1037 (more wall cells were light- or sky-blue-coloured) (Figure 2C,D) than the indehiscent and stringless PHA0595 (navy-blue-coloured cells) (Figure 2A,B).

**Figure 3:**
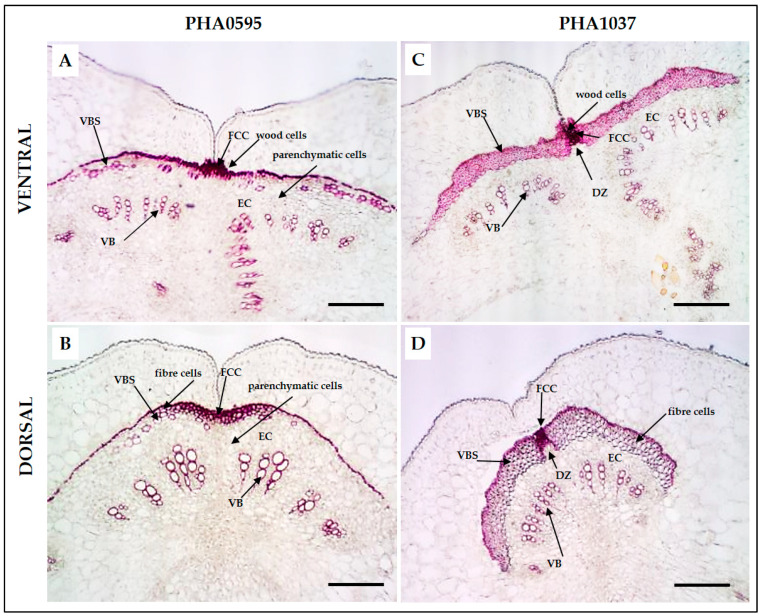
General overview of Phloroglucinol HCl (Wiesner staining)-stained pod sutures in transverse sections of common bean. Paraffin sections (20 μm thick) of 30 days after anthesis (DAA) pods (**A**–**D**). Transverse pod sections of the ventral suture (**A**–**C**) and dorsal suture (**B**,**D**) of the stringless and dehiscence-resistant PHA0595 genotype (**A**,**B**) and dehiscence-susceptible genotype and stringy genotype PHA1037 (**C**,**D**). This stain is lignin-specific and marks the lignified layer of the valve margin, the endocarp layer, and cells of the vascular bundle. Ventral suture: VS, dorsal suture: DS, bundle cap: BC, dehiscence zone: DZ (in PHA1037), fibre cap cells: FCCs, vascular bundles: VBs, vascular bundle sheath: VBS, epidermis: EP, endocarp: EC, mesocarp: MC. Scale bars: 100 μm.

Lignin deposition was detected using Phloroglucinol staining (Figure 3A–D), specific for lignin-associated hydroxycinnamaldehyde [48], which is a complex organic polymer that is important for wall cells and can also be found between cells and in the cells of all vascular plants [49]. The outer layers, namely, the EP and outer MC, were composed of non-lignified parenchymatous cells in both genotypes. Only the inner MC, which was composed of parenchymatous cells, and the EC, which was composed of thick-walled sclerenchymatous cells, were lignified. There was a single layer of sclerenchymatous cells in PHA0595, which was dehiscence-resistant and stringless (Figure 3A,B). In contrast, PHA1037 had two or three layers (Figure 3C,D) and was dehiscence-susceptible and stringy. Higher lignification was seen for both ventral (Figure 3C) and dorsal (Figure 3D) sutures of the dehiscent and stringy PHA1037 compared to the corresponding tissues of the indehiscent and stringless PHA0595 (Figure 3A,B). Thus, dehiscence-resistance and the absence of string seem to be associated with low levels of lignification of pod valves. Furthermore, the suture in the transverse section consisted of a layer of cells intercalated between the valves, which was almost totally non-lignified, forming an abscission (separation) layer in the dehiscence-susceptible PHA1037, but which was almost absent in the dehiscence-resistant PHA0595. This modification is potentially involved in preventing the opening of the valves. The walls of the cells that surrounded this abscission zone in the ventral suture were heavily thickened in the dehiscent and stringy PHA1037 (Figure 3C) compared to the indehiscent PHA0595 (Figure 3A), which may increase the amount of mechanical tension in the ventral suture to thus facilitate the breakdown and pod dehiscence.

### 2.2. Identification of the Dehiscent Structures Underlying Spatial and Temporal Fruit Development

**Figure 4:**
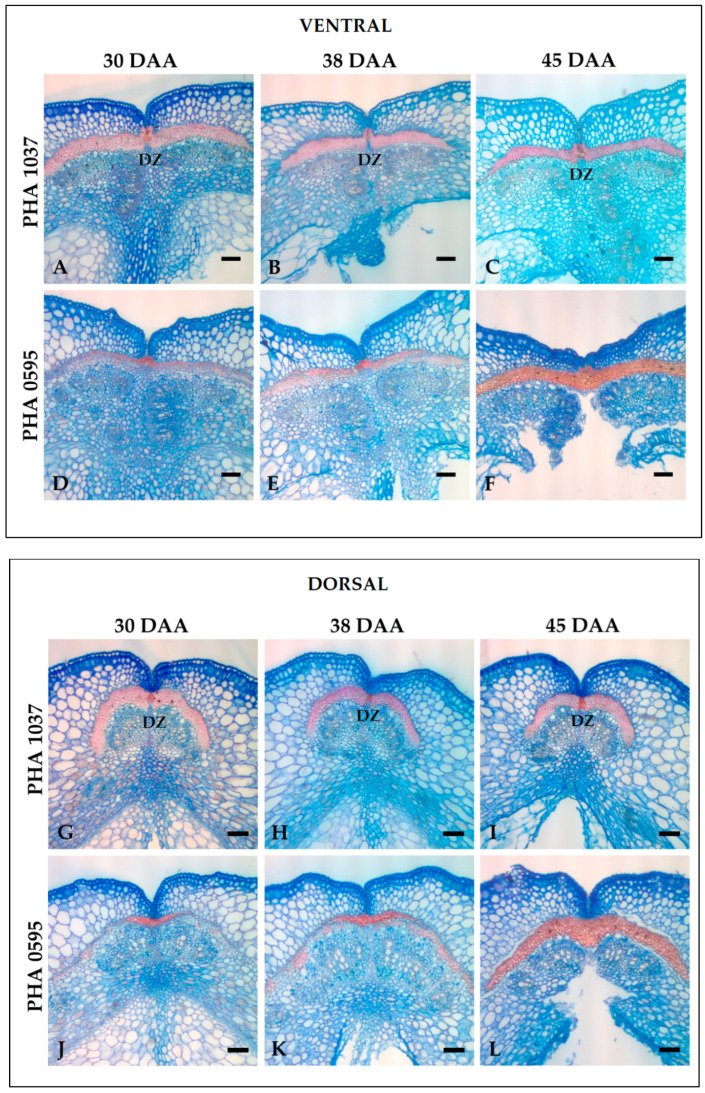
Detection of the abscission layer through common bean pod maturation. Cross-sections of the ventral (**A**–**F**) and dorsal (**G**–**L**) sutures stained with Safranin-O (red staining) and Alcian (blue) in the dehiscent and stringy PHA1037 (**A**–**C**,**G**–**I**) and indehiscent and stringless PHA0595 (**D**–**F**,**J**–**L**). Separation layer and lignified layer cells are present overlying the vascular bundle in PHA1037. Paraffin transversal sections (20 μm) of 30, 38, and 45 days after anthesis (DAA). DZ: dehiscence zone. Scale bars: 100 μm.

Pod cross-section structures from both genotypes were stained with a mixture of Safranin-O, which stains lignified cell walls in red, and Alcian Blue, which stains non-lignified cell walls in blue [19,50], to examine the structures of both sutures across different pod developmental stages (Figure 4A–L).

Microscopic examination of the valve margin regions revealed that the PHA1037 pod began to distinguish a sharp, marked border between valve cells in the ventral (Figure 4A) and dorsal sutures (Figure 4G) at 30 days after anthesis (DAA); however, the border was essentially absent in the pod of the indehiscent PHA0595 (Figure 4D,J). These border or thin-walled separation layer (sl) cells were stained in light blue, and the non-lignified tissues (primary cell walls) were stained in dark blue, while the lignified tissues (with secondary cell walls) were stained in red.

In the PHA1037 pod, lignified valve margin cells were present, overlying the VB, and stained a more intense red with Safranin-O/Alcian Blue than their neighbouring valve cells at 30 DAA in ventral (Figure 4A–C) and dorsal (Figure 4G–I) sutures, and in contrast to PHA0595 (Figure 4D–F,J–L). This variation in stain intensity was also observed with another lignin stain, namely, Phloroglucinol, which was previously used (Figure 3A–D). Observations of the stained sections of PHA1037 did not show marked differences in the cell wall thickness over time (from 30 to 45 DAA). This would indicate early gene regulation for the lignified tissue formation and for the key FCC and DZ structures involved in the dehiscence mechanism.

However, the completely indehiscent PHA0595 showed complete lignification overlying the VBS at 45 DAA (Figure 4D–F,J–L), corresponding to the senescent pod, which greatly increased from 38 to 45 DAA. Our study describes differences between dehiscent and indehiscent genotypes associated with the deposition of lignin, which are in conjunction with spatial and temporal fruit development and agree with other studies [51].

### 2.3. Association of Fruit Dehiscent Structures with Autofluorescence through Pod Maturation

**Figure 5:**
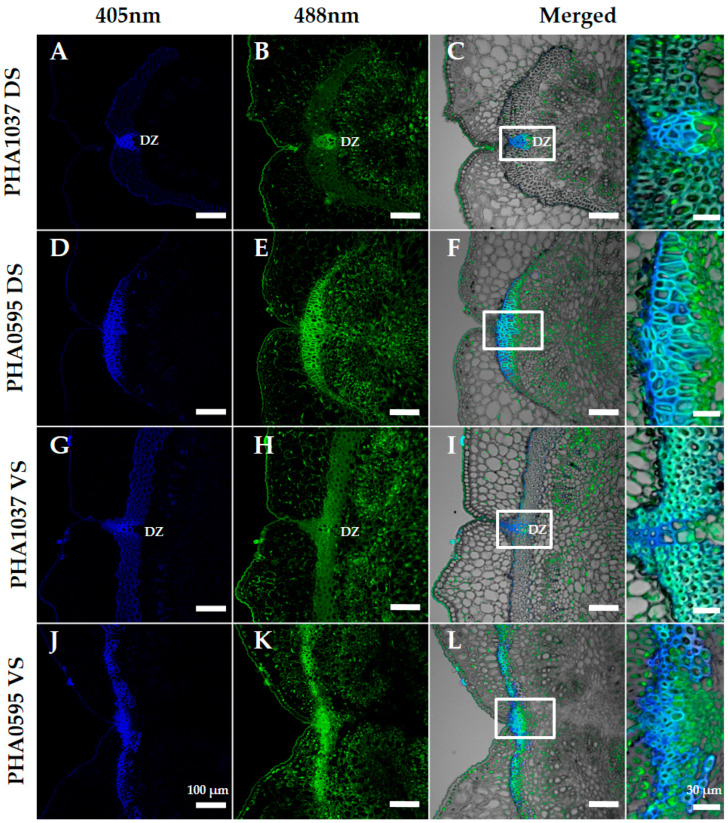
Transverse sections of dorsal (**A**–**F**) and ventral (**G**–**L**) sutures in the dehiscent and stringy genotype PHA1037 (**A**–**C**,**G**–**I**) and indehiscent and stringless genotype PHA0595 (**D**–**F**,**J**–**L**) at 45 days after anthesis (DAA). Excitation at 405 nm with emissions in the range of 400–475 nm (blue, first column), and excitation at 488 nm with emissions in the range of 500–545 nm (green, second column). Images for each channel are maximal projections of five confocal optical sections. The merging of both fluorescent channels is shown in the third and fourth columns, where the bright field was used for anatomical reference. The fourth column shows an optical magnification of the central region of the sutures (white squares in the third column). Suture autofluorescence is patently higher in the indehiscent PHA0595 (**D**–**F**,**J**–**L**) compared to the dehiscent PHA1037 (**A**–**C**,**G**–**I**). Secondary cell wall modifications were clearly different between both genotypes in unstained 20-micron microtome sections excited at 405 nm and 488 nm and comparable to differences shown by other more time-consuming traditional staining methods used, such as Phloroglucinol-HCl, Safranin-O/Alcian Blue, and Toluidine Blue O protocols. Ventral suture: VS, dorsal suture: DS, dehiscence zone: DZ. Scale bars, 100 μm in panoramic views and 30 μm in the magnified regions.

We investigated the association of autofluorescence (in unstained microtome sections excited at 405 and 488 nm) with the structure of the pod sutures through 30 to 45 DAA in both genotypes (dehiscent stringy vs. indehiscent stringless). Figure 5 shows transverse sections of the ventral and dorsal sutures of both genotypes under a confocal laser microscope. Confocal microscopy examination revealed the presence of fluorescent substances in the sutures that were clearly different between both genotypes and comparable to the differences shown by the other more time-consuming traditional staining methods previously used (Phloroglucinol-HCl, Safranin/Alcian Blue, and TBO protocols). We observed two main autofluorescence emission maxima—namely, in the blue (400–475 nm) and green (500–545 nm) regions of the spectrum.

In general, our study showed the maximum of blue and green autofluorescence, which may reflect the content of lignin, in the sclerenchyma cells of the FCC layer at the DZ in the stringy PHA1037 genotype, in both dorsal (Figure 5C) and ventral sutures (Figure 5I). A weaker autofluorescence signal was observed in the rest of the suture parenchyma cells. In the stringless and indehiscent PHA0595, the blue and green autofluorescence signal was maximal along the suture in comparison with the DZ (Figure 5F,L). The bright autofluorescence signal, after excitation in the blue spectral region, in the FCC layer at the DZ correlated well with the dehiscent phenotype of PHA1037 at both the dorsal and ventral sutures. This result suggests that autofluorescence could be an indicator of the abscission layer development at pod sutures and may provide a means for the non-destructive assessment of the dehiscent phenotype.

**Figure 6:**
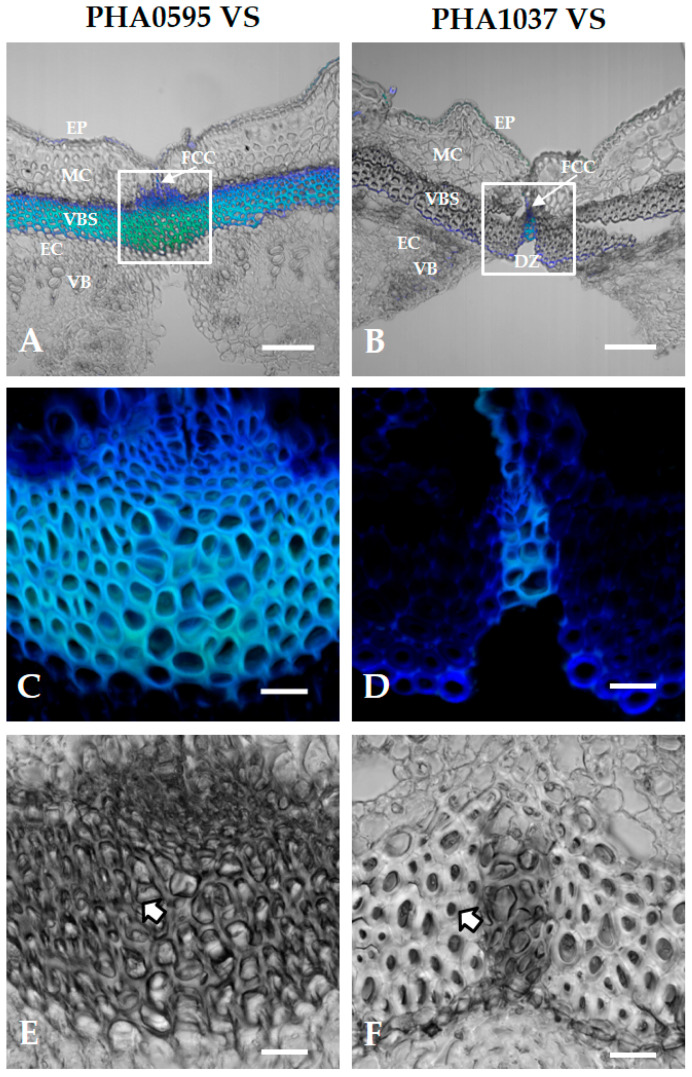
Anatomical cell wall comparison of ventral suture at 45 days after anthesis (DAA) of stringless PHA0595 and stringy PHA1037 genotypes. Representative confocal images at the ventral suture level show that autofluorescence is patently higher in the stringless PHA0595 (**A**) compared to the stringy PHA1037 (**B**). (**C**,**D**) Optical magnification of the central region of the sutures (white boxes in A and B) also shows differences in cell wall thickness. Suture autofluorescence levels in the stringless ventral suture (**C**) are higher than in the stringy suture (**D**), presumably due to different lignin deposition and other secondary wall components. (**E**,**F**) Transmitted light images of the same regions in (**C**,**D**) show thicker and round-shaped cell walls in the stringy genotype (**F**), whereas those in the stringless genotype (**E**) tend to be thinner and polyhedric (see arrows). Only the dehiscence zone (DZ) in the stringy genotype shows this same pattern (higher autofluorescence and thinner, polyhedric cell walls). Confocal images are maximum projections of five optical sections. Ventral suture: VS, dehiscence zone: DZ, fibre cap cells: FCCs, vascular bundles: VB, vascular bundle sheath: VBS, epidermis: EP, endocarp: EC, mesocarp: MC. Scale bars: 100 μm in (**A**,**B**), and 20 μm in (**C**–**F**).

As already mentioned, there were prominent VBSs in both dorsal and ventral sutures of the pod (Figure 1G), which consisted of several layers of small cells with few intercellular spaces (Figure 2A–D). According to the literature [52], this VBS is composed of three types of cells, namely, parenchyma, wood, and sclerenchyma cells (fibres), and these last cells are mainly responsible for the strength of the string. The two genotypes examined were anatomically distinct at the ventral suture in terms of their shape and organization of the three cell types (Figure 6). The area around the ventral suture contained many of the same features as those we found at the dorsal suture, and for this reason, we only present here the ventral suture. The PHA0595 genotype contained numerous intercellular spaces and intact cell corners (Figure 6C,E). Cell walls tended to be thinner and polyhedric. In the PHA1037 genotype (Figure 6D,F), the intercellular spaces were absent, and the secondary cell wall had a well-defined layer extending around the circumference of the cell. Cell walls were of an average thickness and showed some degree of rounding.

## 3. Discussion

Histochemical methods may sometimes present limitations, especially regarding their application in breeding programs for studying various genotypes grown in different environments, because of their complexity, and they are frequently time-consuming. The development of highly efficient and simple techniques therefore represents a major breakthrough. Here, we have extended the usefulness of autofluorescence by demonstrating that it may resolve anatomical distinctions of components, similarly to a large number of classical histochemical dyes, and allow high-quality imaging of primary and secondary wall components of the common bean pod to be achieved.

Legume fruit samples, and specifically suture sections, are often difficult to visualize due to tissue complexity. To improve this, we demonstrated the reliability of a fast and low-cost method in which paraffin sections of ventral and dorsal sutures can be efficiently visualized without staining and in combination with various fluorescent excitation and emission wavelength ranges. This rapid autofluorescence method provides good-quality images and is applicable to identify secondary cell wall modifications, which can facilitate the study of pod dehiscence and the pod string in the common bean.

In our work, the maximum signal of suture autofluorescence in the sclerenchyma cells of the FCC layer at the DZ, at 45 DAA of pod maturation, correlates well with the secondary cell wall modifications of the dehiscent and stringy genotype at the ventral suture. According to data in the literature [40], blue-light-induced green autofluorescence in the range of 500–545 nm can be explained by the presence of lignin. The authors of [53] reported a negative association between dehiscence resistance and the degree of lignification of the connection of the valves with the replum in brassica crops. Previous studies [51,54] have shown that proper lignification in some species, such as legumes, results in the hardening of the fruits during development and causes major cell wall modifications in which certain cell types and tissues are formed. The autofluorescent lignin molecule is the dominant chemical component, in comparison with the other cell wall components (i.e., hemicellulose and cellulose) in the dehiscent pods of the common bean [33]. In fact, major lignification in the DZ was reported in soybeans [28], rapeseeds [18], and common beans [4,33,55] to confer dehiscence resistance. This appears to be a potentially useful selection criterion for evaluating dehiscence-resistant genotypes in breeding material [56], and it may suggest that bright autofluorescence is caused by such chemicals.

The dehiscence-resistant and stringless genotype presented a larger VB area and larger FCCs, and external valve margin cells were significantly thicker than in the stringy and dehiscence-susceptible genotype. Our results also suggest that the cells in the VB area showed some degree of rounding in shape and were arranged more closely with less interstitial substance in the dehiscence-susceptible genotype. Other studies observed marked differences associated with the dehiscent phenotype related to the geometrical arrangement of the lateral cell wall [57], indicating compression wood severity [58]. Regarding this area, it was found to be larger in dehiscence-susceptible soybeans [59], while in common vetches, it was larger in the dehiscence-resistant plants [60]. This difference may be explained by the distant evolutionary relationships between the soybean and common vetch, which belong to the phaseoloid and inverted-repeat-lacking clades, respectively, in the papilionoid phylogeny tree [61].

An abscission (separation) layer, with thicker walls of the surrounding cells, was found in the dehiscence-susceptible genotype, and it was almost absent in the dehiscence-resistant genotype. The absence of this layer in the dehiscence-resistant genotype gives rise to a greater cohesive force connecting both valves, making it more difficult to separate the two valves, and thus allows for pod dehiscence. In the evolution of the fruit structure in the Brassicaceae, the loss of dehiscence was associated with the abortion or failure to develop an abscission layer in its fruit [18]. More recently, Vittori et al. [55] suggested that a non-functional abscission layer could be responsible for the loss of pod dehiscence in the common bean, and Parker et al. [62] found in stringy genotypes that the vascular sutures have a strong secondary thickening and 3–6 fibre cell layers, excluding the DZ. This is in agreement with our observations—namely, the presence of a clear abscission layer in the dehiscent genotype but a lack of this layer in the non-dehiscent genotype. This suggests that the lack of an abscission layer may be due to ectopic lignification, as a key structure to prevent dehiscence [13,55,60,63], and is likely due to selection against dehiscence during the domestication process.

## 4. Materials and Methods

### 4.1. Plant Growth and Phenotype Evaluation

Plants of the landrace PHA1037 (nuña bean) and the cultivar PHA0595 (Bolita) were grown in a growth chamber (20–25 °C, relative humidity 70–90%, 8 h day/16 h night short day (SD) photoperiod regime). PHA0595 is a Spanish improved line, which shows an indehiscent and stringless pod (Figure 1A–C). PHA1037 is a landrace from Bolivia that possesses dehiscent and stringy pods (Figure 1D,E). Ninety flowers on eighteen plants *per* genotype were tagged at flower anthesis, and pods were collected at R8 (pod fill), corresponding to 30 to 45 days after flower anthesis (DAA), and R9 (pod maturation) stages [45]. For evaluating the dehiscence and presence of the string, two different conditions were applied. Under natural conditions, mature full-sized pods (R8 at 30 DAA) were broken to analyse the pod string phenotype on a scale from 0 to 9, where 0 = stringless, 3 = a few strings, 5 = moderately stringy, 7 = very stringy, and 9 = highly stringy. Under experimental conditions, 50 dried physiological pods (R9 stage at 90 DAA) were collected and kept in an oven at 60 °C for 7 days, and then the number of dehiscent pods was counted in order to determine the pod dehiscence percentage [64]. Pod dehiscence was measured as the percentage of open pods using a scale of 1 to 5, where 1 = 0%, 2 = 1–10%, 3 = 11–25%, 4 = 26–50%, and 5 = > 50% dehiscent [65]. The statistics were an average of six biological replicates per genotype. 

### 4.2. Sample Preparation, Tissue Embedding, Blocking, and Sectioning

The pod structures of PHA1037 and PHA0595 were observed with transversely hand-cut sections at the medium zone of mature pod tissue (from 30 to 45 DAA). Thick sections (0.8–1.0 cm thick) were cut in transverse segments corresponding with the ventral and dorsal sutures of the pod (Figure 1F,G). Samples were immediately fixed with a fresh 4 °C FAE solution (37% (*w*/*v*) Formaldehyde, 5% Acetic Acid, 100% EtOH, and 70% (*v*/*v*) Glutaraldehyde) with points of vacuum treatment to improve penetration of the fixative for 3 h and then kept at 4 °C.

For epoxy resin embedding, samples were dehydrated in an ethanol series and embedded in EPON812 (Electron Microscopy Sciences) according to the manufacturer’s instructions. Semithin sections (0.5–1.0 μm thick) were obtained with a Leica Ultracut R ultramicrotome using a Diatome diamond knife and attached to clean glass slides. Sections were stained with Toluidine blue O–Borax (1%) on a hot plate set at 70 °C for 2 min, mounted with DPX (VWR Prolabo), and observed with a conventional light microscope (Olympus BX51 equipped with a DP50 digital camera). 

For paraffin embedding, samples were dehydrated in an ethanol series, cleared in Histoclear (Apes Scientific), and infiltrated with liquid paraffin (HistoComp, Casa Álvarez) using an automatic tissue processor (Leica TP1020). Pod samples embedded in paraffin blocks were cut into 20 μm thick sections using a Leica RM2125 rotary microtome, floated on a 45 °C water bath, and transferred to Polysine adhesion slides (Menzel GmbH and Co KG, Braunschweig, Germany). Sections were allowed to dry overnight at 37 °C and stored at room temperature until use.

### 4.3. Histochemical Staining Procedure and Light Microscopy

Prior to staining, paraffin sections were deparaffinized in Histoclear, rehydrated in an ethanol series followed by distilled water, and then used for the following staining procedures:

Phloroglucinol-HCl (Wiesner) staining. Samples were stained in a Phloroglucinol solution (P3502-25G Sigma-Aldrich; 2% Phloroglucinol in 95% ethanol, freshly prepared) for 2 to 3 min, then washed with 50% HCl for 1 min, and observed immediately [17]. Thickened and lignified cell walls were stained purple–red.

TBO Toluidine blue staining. Samples were stained with Toluidine blue O (Panreac, Spain) and Borax (Merck, Spain) (1%) in water at room temperature for 3 min, washed with water for 1 min, and air dried [66]. Cell walls were stained in different shades of blue, and some metachromasia could be observed at the suture level (secondary cell walls) compared to primary cell walls. 

Safranin-O/Alcian Blue staining. Samples were first stained with Safranin-O (Sigma-Aldrich; 1% dissolved in water) for 1 min, washed with water four times, and then stained for 5 min in a 1% Alcian Blue solution at pH 2.5 (Merck, Spain) and mounted with 70% glycerol in PBS [67,68].

Stained sections were observed under a conventional light microscope (Nikon Eclipse E200 equipped with an AxioCam ERc5s digital camera).

### 4.4. Confocal Microscopy

Unstained 20-micron-thick paraffin sections were deparaffinized and rehydrated as previously described, mounted in 70% glycerol, and observed under a laser scanning confocal microscope (Nikon A1 confocal microscope). Samples were sequentially excited with 2 laser lines, 405 nm and 488 nm, and autofluorescence images were acquired with a 20× water multi-immersion objective. Confocal images were the maximum fluorescence projection of 5 optical sections taken with a 2-micron Z-step interval.

## 5. Conclusions

In this study, we compared different histochemical staining and autofluorescence (excitation at 405 and 488 nm) methods to determine the association with the dehiscence and string of the pod and the anatomy of the ventral and dorsal sutures in two genotypes of the common bean displaying differential susceptibilities to dehiscence. The high degree of dehiscence resistance appears to be due to the lack of an abscission layer in the region of attachment of the valves of the fruit, which is normally found in the sutures of the valves in the dehiscence-susceptible genotype. The layer of cells intercalated between the valves in the dehiscence-susceptible genotype was almost totally non-lignified, and this layer helped to open both sutures and as a consequence, aided the dehiscence process. Moreover, it is important to highlight to what extent early anatomical pod events were affected in similar ways by the domestication event (dehiscence), resulting in the phenotypic pod complexity that can now be seen. The results suggest that the use of autofluorescence imaging approaches, such as confocal microscopy, represents a simpler and faster method for studying the DZ and the secondary cell wall structure of the pod sutures in the common bean, clearly differentiating dehiscence-contrasting genotypes and thereby providing a considerable advantage over other histochemical protocols.

## Data Availability

Not applicable.

## References

[1] Doebley J.F., Gaut B.S., Smith B.D. (2006). The molecular genetics of crop domestication. Cell.

[2] Purugganan M., Fuller D.Q. (2009). The nature of selection during plant domestication. Nature.

[3] Parker T.A., Sassoum L., Gepts P. (2021). Pod shattering in grain legumes: Emerging genetic and environment-related patterns. Plant Cell.

[4] Parker T.A., Berny Mier T., Palkovic A., Jernstedt J., Gepts P. (2020). Pod indehiscence is a domestication and aridity resilience trait in common bean. New Phytol..

[5] Zhang Q., Tu B., Liu C., Liu X. (2018). Pod anatomy, morphology and dehiscing forces in pod dehiscence of soybean (*Glycine max* (L.) Merrill). Flora.

[6] Lush W.M., Evans L.T. (1981). The domestication and improvement of cowpeas (*Vigna unguiculata* (L.) WALP.). Euphytica.

[7] Gepts P., Debouck D.G., Voysest O., Van Schoonhoven A. (1991). Origin, domestication, and evolution of the common bean, *Phaseolus vulgaris*. Common Beans: Research for Crop Improvement.

[8] Romkaew J., Nagaya Y., Goto M., Suzuki K., Umezaki T. (2008). Pod dehiscence in relation to chemical components of pod shell in soybean. Plant Prod. Sci..

[9] Prakken R. (1934). Inheritance of colours and pod characters in *Phaseolus vulgaris* L.. Genetica.

[10] Koinange E.M.S., Singh S.P., Gepts P. (1996). Genetic control of the domestication syndrome in common bean. Crop Sci..

[11] Kongjaimun A., Somta P., Tomooka N., Kaga A., Vaughan D.A., Srinives P. (2013). QTL mapping of pod tenderness and total soluble solid in yardlong bean [*Vigna unguiculata* (L.) Walp. subsp. *unguiculata* cv. -gr. *sesquipedalis*]. Euphytica.

[12] Suanum W., Somta P., Kongjaimun A., Yimram T., Kage A., Tomooka N. (2016). Co-localization of QTLs for pod fiber content and pod shattering in F_2_ and backcross populations between yardlong bean and wild cowpea. Mol. Breed..

[13] Takahashi Y., Kongjaimun A., Muto C., Kobayashi Y., Kumagai M., Sakai H., Satou K., Teruya K., Shiroma A., Shimoji M. (2020). Same Locus for Non-shattering Seed Pod in Two Independently Domesticated Legumes, *Vigna angularis* and *Vigna unguiculata*. Front. Genet..

[14] Ogutcen E., Pandey A., Khan M.K., Marques E., Penmetsa R.V., Kahraman A., von Wettberg E.J. (2018). Pod shattering: A homologous series of variation underlying domestication and an avenue for crop improvement. Agronomy.

[15] Di Vittori V., Gioia T., Rodriguez M., Bellucci E., Bitocchi E., Nanni L., Attene G., Rau D., Papa R. (2019). Convergent evolution of the seed shattering trait. Genes.

[16] Spence J., Vercher Y., Gates P., Harris N. (1996). ‘Pod shatter’ in *Arabidopsis thaliana*, *Brassica napus* and *B. juncea*. J. Microsc..

[17] Balanzà V., Roig-Villanova I., Di Marzo M., Masiero S., Colombo L. (2016). Seed abscission and fruit dehiscence required for seed dispersal rely on similar genetic networks. Development.

[18] Zhang Y., Shen Y.Y., Wu X.M., Wang J.B. (2016). The basis of pod dehiscence: Anatomical traits of the dehiscence zone and expression of eight pod shatter-related genes in four species of Brassicaceae. Biol. Plant..

[19] Meakin P.J., Roberts J.A. (1990). Dehiscence of fruit in oilseed rape (*Brassica napus* L.) II. The role of cell wall degrading enzymes and ethylene. J. Exp. Bot..

[20] Østergaard L., Borkhardt B., Ulvskov P., Roberts J.A., Gonzalez-Carranza Z.H. (2007). ’Dehiscence’ in Plant Cell Separation and Adhesion.

[21] Morgan C.L., Bruce D.M., Child R., Ladbrooke Z.L., Arthur A.E. (1998). Genetic variation for pod shatter resistance among lines of oilseed rape developed from synthetic *B. napus*. Field Crops Res..

[22] Christiansen L.C., Dal Degan F., Ulvskov P., Borkhardt B. (2002). Examination of the dehiscence zone in soybean pods and isolation of a dehiscence-related endopolygalacturonase gene. Plant Cell Environ..

[23] Østergaard L., Kempin S.A., Bies D., Klee H.J., Yanofsky M.F. (2006). Pod shatter-resistant Brassica fruit produced by ectopic expression of the *FRUITFULL* gene. Plant Biotechnol. J..

[24] Tiwari S., Bhatia V. (1995). Characters of pod anatomy associated with pod shattering in soybean. Ann. Bot..

[25] Ferrándiz C. (2002). Regulation of fruit dehiscence in Arabidopsis. J. Exp. Bot..

[26] Yang J.B., Wright R.L., McGraw R.L. (1990). Seed pod dehiscence in birdsfoot trefoil, *Lotus conimbricensis*, and their interspecific somatic hybrid. Can. J. Plant Sci..

[27] Fourquin C., del Cerro C., Victoria F.C., Vialette-Guiraud A., de Oliveira A.C., Ferrandiz C. (2013). A change in SHATTERPROOF protein lies at the origin of a fruit morphological novelty and a new strategy for seed dispersal in the Medicago genus. Plant Physiol..

[28] Dong Y., Yang X., Liu J., Wang B.H., Liu B.L., Wang Y.Z. (2014). Pod dehiscence resistance associated with domestication is mediated by a *NAC* gene in soybean. Nat. Commun..

[29] Dong Y., Wang Y.Z. (2015). Seed shattering: From models to crops. Front. Plant Sci..

[30] Li L.F., Olsen K.M. (2016). To Have and to Hold: Selection for Seed and Fruit Retention During Crop Domestication. Curr. Top. Dev. Biol..

[31] Ballester P., Ferrándiz C. (2017). Shattering fruits: Variations on a dehiscent theme. Curr. Opin. Plant Biol..

[32] Funatsuki H., Suzuki M., Hirose A., Inaba H., Yamada T., Hajika M., Komatsu K., Katayama T., Sayama T., Ishimoto M. (2014). Molecular basis of a shattering resistance boosting global dissemination of soybean. Proc. Natl. Acad. Sci. USA.

[33] Murgia M.L., Attene G., Rodriguez M., Bitocchi E., Bellucci E., Fois D., Nanni L., Gioia T., Albani D.M., Papa R. (2017). A Comprehensive Phenotypic Investigation of the “Pod-Shattering Syndrome” in Common Bean. Front. Plant Sci..

[34] Morgan C.L., Ladbrooke Z.L., Bruce D.M., Child R., Arthur A.E. (2000). Breeding oilseedrape for pod shattering resistance. J. Agric. Sci..

[35] Davies G.C., Bruce D.M. (1997). Fracture mechanics of oilseed rape pods. J. Mater. Sci..

[36] Dong D., Dong R., Wang Y., Nie B., Liu Z. (2016). Study on pod development and ventral suture structure of *Vicia sativa* cultivar Lanjian NO. 3. Acta Bot. Boreali-Occident. Sin..

[37] Tsuchiya T. (1987). Physiological and genetic analysis of pod shattering in soybean. Jarq-Jpn. Agric. Res. Q..

[38] Suzuki M., Fujino K., Funatsuki H. (2009). A major soybean QTL, *qPDH1*, controls pod dehiscence without marked morphological change. Plant Prod. Sci..

[39] Boerjan W., Ralph J., Baucher M. (2003). Lignin biosynthesis. Annu. Rev. Plant Biol..

[40] Donaldson L. (2020). Autofluorescence in Plants. Molecules.

[41] Donaldson L.A. (2001). Lignification and lignin topochemistry—An ultrastructural view. Phytochemistry.

[42] Pesquet E., Ranocha P., Legay S., Digonnet C., Barbier O., Pichon M., Goffner D. (2005). Novel markers of xylogenesis in zinnia are differentially regulated by auxin and cytokinin. Plant Physiol..

[43] Liljegren S. (2010). Phloroglucinol stain for lignin. Cold Spring Harb. Protoc..

[44] Tolivia D., Tolivia J. (1987). Fasga: A new polychromatic method for simultaneous and differential staining of plant tissues. J. Microsc..

[45] Fernández de Córdova F., Gepts P., López M. (1986). Etapas de Desarrollo de la Planta de Fríjol Común (Phaseolus vulgaris L.).

[46] Debouck D.G., Hidalgo R. (1985). Morfología de la planta de frijol común. Frijol: Investigación y Producción.

[47] Carlson J.B., Lersten N.R., Shibles R.M., Harper J.E., Wilson R.F., Shoemaker R.C. (2004). Reproductive morphology. Soybeans: Improvement, Production, and Uses.

[48] Bonawitz N.D., Kim J.I., Tobimatsu Y., Ciesielski P.N., Anderson N.A., Ximenes E., Maeda J., Ralph J., Donohoe B.S., Ladisch M. (2014). Disruption of Mediator rescues the stunted growth of a lignin-deficient Arabidopsis mutant. Nature.

[49] Vanholme R., Demedts B., Morreel K., Ralph J., Boerjan W. (2010). Lignin biosynthesis and structure. Plant Physiol..

[50] Graham E.T., Trentham W.R. (1998). Staining paraffin extracted, alcohol rinsed plant tissue with an aqueous mixture of three dyes. Biotechnol. Histochem..

[51] Rogers L.A., Campbell M.M. (2004). The genetic control of lignin deposition during plant growth and development. New Phytol..

[52] Drijfhout E. (1970). Influence of temperature on string formation of beans (*Phaseolus vulgaris*). Euphytica.

[53] Thurling N., Howieson J. (1982). Genotypic variation in shattering resistance in spring rape. Australas. Plant Breed. Genet. Newsl..

[54] Liljegren S., Ditta G., Eshed Y., Savidge B., Bowman J.L., Yanofsky M.F. (2000). *SHATTERPROOF MADS*-box genes control seed dispersal in Arabidopsis. Nature.

[55] Di Vittori V., Bitocchi E., Rodriguez M., Alseekh S., Bellucci E., Nanni L., Gioia T., Marzario S., Logozzo G., Rossato M. (2021). Pod indehiscence in common bean is associated to the fine regulation of *PvMYB26* and a non-functional abscission layer. J. Exp. Bot..

[56] Kadkol G.P., Beilharz V.C., Halloran G.M., Macmilla R.H. (1986). Anatomical Basis of Shatter-resistance in the Oilseed Brassicas. Aust. J. Bot..

[57] Aguilar-Benitez D., Rubio J., Millán T., Gil J., Die J.V., Castro P. (2020). Genetic analysis reveals *PDH1* as a candidate gene for control of pod dehiscence in chickpea. Mol. Breed..

[58] Donaldson L.A., Radotic K. (2013). Fluorescence lifetime imaging of lignin autofluorescence in normal and compression wood. J. Microsc..

[59] Tu B., Liu C., Wang X., Li Y., Zhang Q., Liu X., Herbert S.J. (2019). Greater Anatomical Differences of Pod Ventral Suture in Shatter-Susceptible and Shatter-Resistant Soybean Cultivars. Crop Sci..

[60] Jia C., Dong D., Zhou Q., Searle I.R., Liu Z. (2021). Significant cell differences in pod ventral suture in shatter-resistant and shatter-susceptible common vetch accessions. Crop Sci..

[61] Bertioli D.J., Moretzsohn M.C., Madsen H., Sanda N., Leal-Bertioli S.C., Guimaraes P.M., Hougaard B.K., Fredslund J., Schauser L., Nielsen A.M. (2009). An analysis of synteny of *Arachis* with *Lotus* and *Medicago* sheds new light on the structure, stability and evolution of legume genomes. BMC Genom..

[62] Parker T., Cetz J., de Sousa L.L., Kuzay S., Lo S., de Oliveira Floriani T., Njau S., Arunga E., Duitama J., Jernstedt J. (2022). Loss of pod strings in common bean is associated with gene duplication, retrotransposon insertion, and overexpression of *PvIND*. New Phytol..

[63] Dong D., Yan L., Dong R., Liu W., Wang Y., Liu Z. (2017). Evaluation and analysis of pod dehiscence factors in shatter-susceptible and shatter-resistant common vetch. Crop Sci..

[64] Romkaew J., Umezaki T. (2006). Pod dehiscence in soybean: Assessing methods and varietal difference. Plant Prod. Sci..

[65] Zhang J., Singh A.K. (2020). Genetic control and geo-climate adaptation of pod dehiscence provides novel insights into soybean domestication. G3 Genes Genomes Genet..

[66] Mitra P.P., Loque D. (2014). Histochemical staining of *Arabidopsis thaliana* secondary cell wall elements. J. Vis. Exp..

[67] Sessions R.A., Zambryski P.C. (1995). Arabidopsis gynoecium structure in the wild and in ettin mutants. Development.

[68] Zúñiga-Mayo V.M., Marsch-Martinez N., De Folter S. (2012). JAIBA, a class-II HD-ZIP transcription factor involved in the regulation of meristematic activity, and important for correct gynoecium and fruit development in Arabidopsis. Plant J..

